# Heantos-4, a natural plant extract used in the treatment of drug addiction, modulates T-type calcium channels and thalamocortical burst-firing

**DOI:** 10.1186/s13041-016-0274-7

**Published:** 2016-12-05

**Authors:** Stuart M. Cain, Soyon Ahn, Esperanza Garcia, Yiming Zhang, Zeina Waheed, John R. Tyson, Yi Yang, Tran Van Sung, Anthony G. Phillips, Terrance P. Snutch

**Affiliations:** 1 Michael Smith Laboratories and Djavad Mowafaghian Centre for Brain Health, University of British Columbia, 219-2185 East Mall, Vancouver, BC V6T 1Z4 Canada; 2 Department of Psychiatry, University of British Columbia, Vancouver, Canada; 3 Institute of Chemistry, Vietnam Academy of Science and Technology, Hanoi, Vietnam

**Keywords:** Burst firing, Thalamocortical, Thalamus, T-type, Calcium, Epilepsy, Addiction, Heantos-4

## Abstract

Heantos-4 is a refined combination of plant extracts currently approved to treat opiate addiction in Vietnam. In addition to its beneficial effects on withdrawal and prevention of relapse, reports of sedation during clinical treatment suggest that arousal networks in the brain may be recruited during Heantos administration. T-type calcium channels are implicated in the generation of sleep rhythms and in this study we examined whether a Heantos-4 extraction modulates T-type calcium channel currents generated by the Cav3.1, Cav3.2 and Ca3.3 subtypes. Utilizing whole-cell voltage clamp on exogenously expressed T-type calcium channels we find that Heantos inhibits Cav3.1 and Cav3.3 currents, while selectively potentiating Cav3.2 currents. We further examined the effects of Heantos-4 extract on low-threshold burst-firing in thalamic neurons which contribute to sleep oscillations. Using whole-cell current clamp in acute thalamic brain slices Heantos-4 suppressed rebound burst-firing in ventrobasal thalamocortical neurons, which express primarily Cav3.1 channels. Conversely, Heantos-4 had no significant effect on the burst-firing properties of thalamic reticular neurons, which express a mixed population of Cav3.2 and Cav3.3 channels. Examining Heantos-4 effects following oral administration in a model of absence epilepsy revealed the potential to exacerbate seizure activity. Together, the findings indicate that Heantos-4 has selective effects both on specific T-type calcium channel isoforms and distinct populations of thalamic neurons providing a putative mechanism underlying its effects on sedation and on the thalamocortical network.

## Introduction

Opiate dependence is estimated to affect 15 million people worldwide and opiate overdose is believed to result in approximately 69,000 mortalities per year [1]. While pharmacological therapies can be utilized during rehabilitation to reduce cravings (methodone, buprenorphine, suboxone), block of the rewarding effects (naltrexone) or lessen the negative symptoms of opioid withdrawal (anti-emetics, sedatives, antidepressants), relapse rates remain unfortunately high [2, 3]. As such, there is a pressing need to discover and develop new therapies for the treatment of opiate addiction.

Heantos-4, which is the Greek term for “Plants” is a mixture of organic herbs developed in Vietnam and recently approved for the clinical alleviation of withdrawal symptoms in individuals dependent upon opiates [4]. Additionally, preliminary observations indicate that it aids in the reduction in relapse rates. A sedative effect of Heantos has been reported by patients during the first few days of treatment. Mechanistically, there is little current understanding of how Heantos mediates its effects on opioid withdrawal, relapse or sedation. Recent findings show that oral administration of Heantos-4 reduces drug-seeking behaviors in animal models of morphine addiction [5]. Further, microdialysis experiments have revealed that oral administration of Heantos in rats enhances dopamine efflux in the nucleus accumbens, providing a mechanistic correlate for its effects on addiction [5]. The current study examines the effects of Heantos on burst-firing of thalamic neurons due to the involvement of this brain region in non-REM sleep and the control of arousal [6].

Voltage-gated calcium channels are a class of membrane bound proteins that regulate the cellular entry of calcium ions upon depolarization [7]. T-type calcium channels (Ca_V_3.1- Ca_V_3.3) are a sub-class of calcium channels that activate at more hyperpolarized membrane potentials than their High Voltage-Activated (HVA) counterparts (Ca_V_1.1- Ca_V_1.4 and Ca_V_2.1- Ca_V_2.3). As a result, T-type calcium channels open in response to smaller depolarizations than HVA calcium and sodium channels, endowing them with a unique the ability to modulate cellular excitability at near-resting membrane potentials [8]. Low-threshold burst-firing is a neuronal firing mode wherein a short duration of high frequency action potentials occur upon the crest of a “Low Threshold calcium Spike” (LTS) generated by T-type calcium currents [8, 9]. Thalamic neurons display a well-characterized switch between burst- and tonic-firing depending on a combination of resting membrane potential and excitatory/inhibitory input [10]. While burst-firing occurs under normal conditions in the brain, in particular during sleep [6, 11] it is also associated with pathophysiological neuronal disorders, such as epilepsy [12–14].

In this study we examined whether Heantos-4 can directly modulate exogenously expressed T-type calcium channels (Ca_V_3.1- Ca_V_3.3). Further, we correlated the effects observed for cloned channels with Heantos-mediated modulation of ventrobasal (VB) and reticular thalamic nucleus (TRN) neurons in acute rat brain slices. Finally, we examined the effects of Heantos-4 oral administration on seizure activity in the absence epilepsy model, Genetic Absence Epilepsy Rats from Strasbourg (GAERS).

## Results

### Heantos differentially modulates T-type calcium channel subtypes

**Fig. 1:**
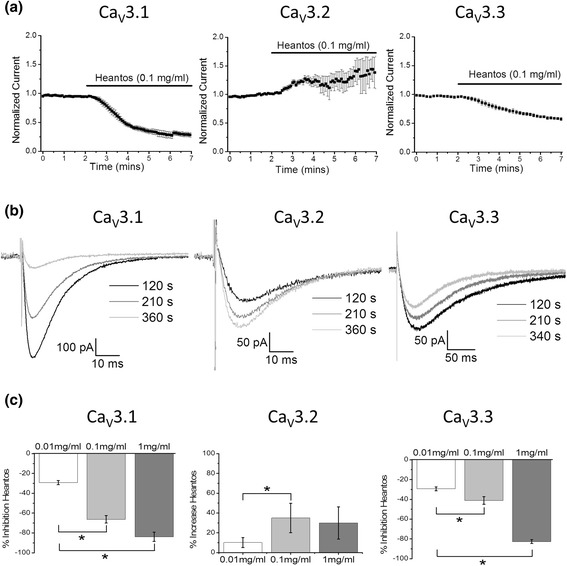
Heantos differentially modulates current density of T-type calcium channel isoforms. **a** Timecourse of action of Heantos (0.1 mg/ml) on individual T-type calcium channel isoforms (*n* = 5 per T-type isoform) exogenously expressed in HEK293 cells. **b** Representative traces of Ca_V_3.1, Ca_V_3.2 and Ca_V_3.3 T-type calcium currents before and after Heantos application. **c** Histograms summarizing concentration-dependent action of Heantos on individual T-type calcium channel isoforms

To assess whether Heantos directly alters T-type calcium channel currents, individual Ca_V_3.1, Ca_V_3.2 and Ca_V_3.3 isoforms were exogenously expressed in HEK293 cells. Following a 5 min application of Heantos-4 extracted in aCSF (0.1 mg/ml, see Methods) Ca_V_3.1 and Ca_V_3.3 currents were significantly inhibited from the baseline (Ca_V_3.1 = −66.2 ± 3.8%, *P* < 0.001 paired T-test; Ca_V_3.3 = −41.2 ± 3.9% *P* < 0.05 paired T-test), whereas, Ca_V_3.2 currents were significantly potentiated (35.0 ± 14.9%, *P* < 0.05 paired T-test; Fig. 1). Furthermore, a concentration-dependent effect was observed with a lower concentration (0.01 mg/ml) across all three isoforms (*n* = 4), and a higher concentration (1 mg/ml) in the Ca_V_3.1 and Ca_V_3.3 channels (Fig. 1c).

**Fig. 2:**
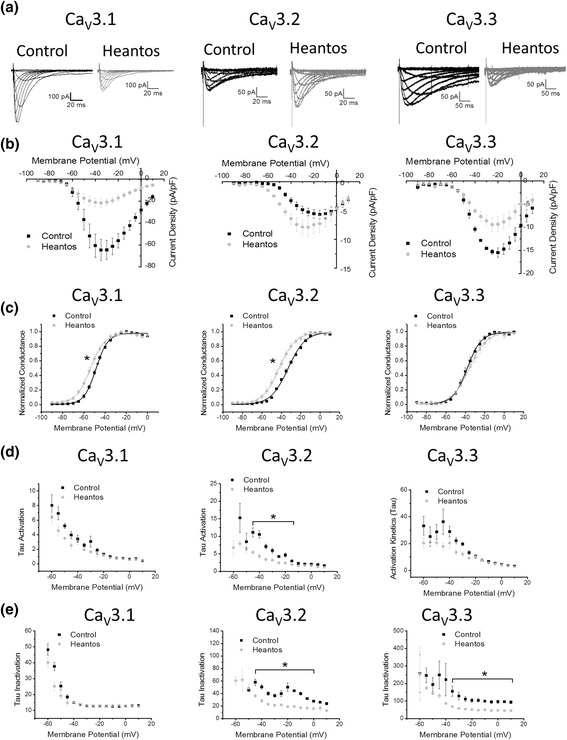
Voltage-dependent effects of Heantos of T-type calcium channel isoforms. **a** Representative current traces to various test potentials from a holding potential of −110 mV before (black) and after (grey) Heantos (0.1 mg/ml). **b** Current density-voltage relationship, **c** voltage dependence of activation, **d** activation kinetics and **e** inactivation kinetics for the effect of Heantos (0.1 mg/ml) on Ca_V_3.1, Ca_V_3.2 and Ca_V_3.3 T-type calcium channel currents (*n* = 5 per isoform)

Voltage-dependence effects of the Heantos-4 were also assessed by examining the current-voltage relationship at the 0.1 mg/ml concentration (Fig. 2). Following Heantos-4 application, a significant leftward shift in the activation curve was observed for both Ca_V_3.1 (V_50_ control = −46.2 ± 2.4 mV, V_50_ Heantos-4 = −51.0 ± 2.9 mV; *P* < 0.05 paired T-test) and Ca_V_3.2 (V_50_ control = 33.3 ± 1.1 mV, V_50_ Heantos-4 = 42.0 ± 1.9 mV; *P* < 0.05 paired T-test), but not for Ca_V_3.3. (V_50_ control = 38.2 ± 1.8 mV, V_50_ Heantos-4 = 37.4 ± .9 mV) (Fig. 2c). In addition, the voltage-dependent kinetics of Ca_V_3.2 currents were also altered by Heantos-4 application with the tau of activation increased between −45 mV and -20 mV and the tau of inactivation increased between −40 mV and 0 mV (Fig. 2d and e). The tau of inactivation for Ca_V_3.3 was significantly smaller (faster inactivating) between −35 mV and 10 mV following Heantos-4 application (Fig. 2e).

### Heantos-4 inhibits burst-firing in VB neurons

**Fig. 3:**
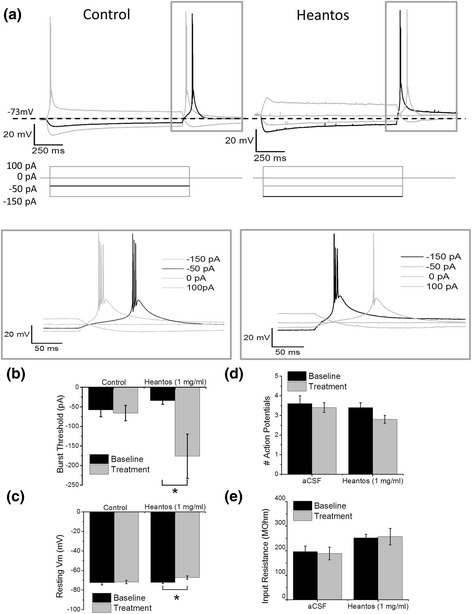
Heantos inhibits burst firing in VB thalamic neurons. **a** Representative voltage traces showing input-output response of the same VB thalamic neuron before (*left*) and after (*right*) application of Heantos (1 mg/ml). Lower panels show current injection protocol. Black traces show the threshold burst voltage and corresponding current injection. The same voltage traces are displayed at a higher time resolution of the region delineated by grey box for clarity. **b** Histograms summarizing mean burst threshold and **c** resting membrane potential before and after Heantos (1 mg/ml) or control (aCSF). **d** Histograms summarizing mean number of action potentials per burst and **e** input resistance for VB thalamic neurons before and after a 5 min application of aCSF (*n* = 5) or Heantos (1 mg/ml (*n* = 5))

Given that Heantos-4 inhibited Ca_V_3.1 and Ca_V_3.3 currents but potentiated Ca_V_3.2 currents, we sought to evaluate its effects in a native system wherein these T-type isoforms are differentially expressed. VB neurons are glutamatergic, sensory thalamocortical neurons that primarily express the Ca_V_3.1 channel [15–17] and we hypothesized that Heantos-4 would suppress burst-firing in this neuronal class. VB neurons can display tonic-firing or rebound burst-firing upon depolarization or hyperpolarization from their resting membrane potential, respectively [9, 18]. If the resting membrane potential is in range wherein the population of T-type calcium channels are balanced in the inactivated and closed states, a LTS can also occur via depolarization (as shown in Fig. 3a). For the purposes of this study we examined only rebound bursts since not all VB neurons displayed depolarizing bursts.

Current clamp recordings were performed on VB neurons using an acute brain slice preparation (Fig. 3). Rebound burst-firing threshold (≥3 action potentials in 50 ms) was determined by applying incremental current injection steps from the resting membrane potential (Fig. 3a). Heantos-4 had little effect on current required to generate rebound burst-firing at concentrations at or below 0.1 mg/ml (not shown). However, at 1 mg/ml Heantos-4 induced a significant increase the burst threshold (baseline = −34 ± 9.3 pA, Heantos-4 = −176 ± 56.6 pA, *P* < 0.05 paired T-test; Fig. 3a and b) which was not observed in control (aCSF applied) neurons (baseline = −58 ± 17.0, control = −66 ± 19.6; *P* > 0.05, paired T-test). This equated to a significant % increase in burst threshold in Heantos-4 treated neurons compared to control (control = 17.3 ± 17.6%, Heantos-4 = 504.7 ± 136.2%, *P* < 0.05 T-test). Heantos-4 also induced a modest but significant depolarization of the resting membrane potential (baseline = −72.2 ± 1.0 mV, Heantos = −67.2 ± 1.8 mV; *P* < 0.05, paired T-test, Fig. 3c), that was not observed in control neurons (baseline = −72.3 ± 2.3, control = −71.7 ± 1.8; *P* > 0.05, paired T-test). Heantos-4 had no effect on the number of action potentials per burst (Fig. 3d) or on the input resistance of VB neurons (Fig. 3e).

### Heantos-4 does not affect burst-firing in TRN neurons

**Fig. 4:**
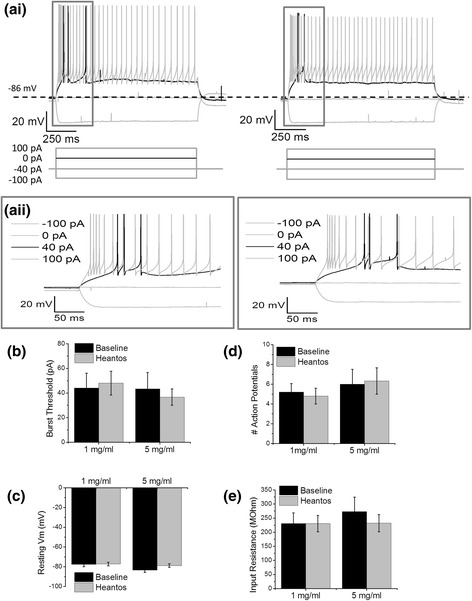
Heantos does not affect burst firing in TRN neurons. ** a** Representative voltage traces showing input-output response of the same TRN thalamic neuron before (left) and after (right) application of Heantos (1 mg/ml, 5 mg/ml). Lower panels show current injection protocol. Black traces show the threshold burst voltage and corresponding current injection. The same voltage traces are displayed at a higher time resolution of the region delineated by grey box for clarity. **b** Histograms summarizing mean current injection for burst threshold, **c** resting membrane potential, **d** number of action potentials and **e** mean input resistance for TRN thalamic neurons before and after a 5 min application of Heantos (1 mg/ml (*n* = 5), 5 mg/ml (*n* = 3))

While VB neurons primarily express Ca_V_3.1 channels, the GABAergic TRN neurons that project to VB neurons express a combination of Ca_V_3.2 and Ca_V_3.3 [15, 17, 19]. Since Heantos-4 potentiates Ca_V_3.2 but inhibits Ca_V_3.3 (Figs. 1 and 2) either a modest inhibition/potentiation or no effect could be hypothesized depending upon the relative co-expression of Ca_V_3.2 and Ca_V_3.3 in TRN neurons. The resting membrane potential of TRN neurons is hyperpolarized in comparison to VB neurons and as a result these neurons burst-fire in response to depolarization, but not hyperpolarization under normal conditions (Fig. 4a).

Current clamp recordings were performed on TRN neurons under the same conditions as described for VB neurons. At a concentration of 1 mg/ml Heantos-4 had no effect on TRN burst-firing threshold or number of action potentials per burst. Similarly, no effect was observed on resting membrane potential or input resistance. A higher concentration of 5 mg/ml Heantos was also tested on TRN neurons. No significant effects were seen on burst firing or passive membrane properties at the 5 mg/ml Heantos concentration (Fig. 4b-e).

### Heantos-4 exacerbates absence seizures in GAERS

Burst-firing has been observed in key epileptogenic neurons in a number of animal epilepsy models [12, 13]. The GAERS absence model displays spontaneous 5–9 Hz Spike-Wave Discharges (SWDs), the electroencephalographic correlate of absence seizures, from a juvenile age that intensify with development [20, 21] and burst-firing in GAERS TRN neurons occurs in a phase-locked manner with SWDs [22]. While a polygenic etiology is believed to underlie seizures in this model, approximately 65-75% of the epileptic phenotype can be attributed to a gain-of-function missense mutation in the domain III-IV linker of the Ca_V_3.2 channel gene [23]. Within the thalamocortical network that drives absence seizures Ca_V_3.2 channels are expressed both in layer V of the cortex and in TRN neurons,[15] and Ca_V_3.2 channels are upregulated in the GAERS model [24, 25]. As such, it is likely that seizures in this model are caused by hyperexcitable cortico-reticular burst-firing.

**Fig. 5:**
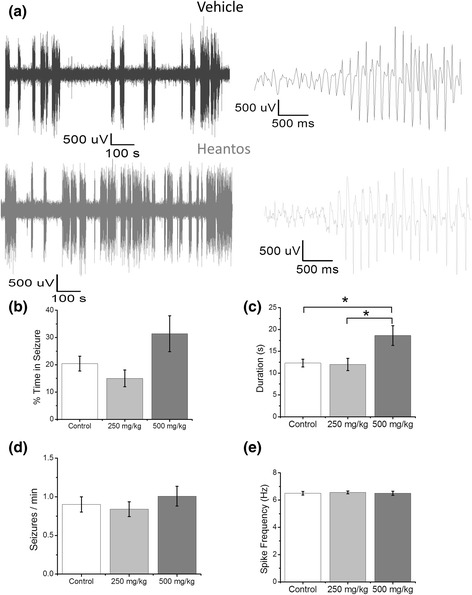
Heantos exacerbates seizures in the GAERS model. **a** Representative EEG traces from GAERS orally administered with control (0.5% CMC; upper traces) or Heantos (500 mg/kg; lower traces) at low (left trace) and high (right traces) time resolution. Histograms displaying mean data for **b** % time spent in seizure state, **c** seizure duration, **d** number of seizures and **e** spike frequency

Heantos-4 in its dry powdered form suspended in CMC/saline (see Methods) was orally administered to GAERS rats 30 min prior to wireless EEG recording and data acquisition for 60 min. Spontaneous seizures occurred throughout the recording period (Fig. 5). Heantos-4 had no effect on any of the seizure parameters measured at the 250 mg/kg dose compared to control animals. However, at a dose of 500 mg/kg there was a significant increase in seizure duration (control = 12.3 ± 0.9 s (*n* = 5), 250 mg/kg = 12.0 ± 1.4 s (*n* = 3), 500 mg/kg = 18.6 ± 2.3 s (*n* = 3); control vs 250 mg/kg *P* = 0.986, control vs 500 mg/kg *P* = 0.004, 250 mg/kg vs 500 mg/kg *P* = 0.011 *ANOVA*); Fig. 5c). We also observed a variable albeit non-significant increase in the % time spent in the seizure state and in the number of seizures following 500 mg/kg Heantos-4 administration (Fig. 5b, d). Conversely, the spike frequency associated with seizures was unaffected by Heantos-4 at either dose (Fig. 5e). Together these findings indicate that at high doses of oral administration Heantos-4 may exacerbate seizure activity in animals with a predisposal to cortico-reticular seizures. Mechanistically in GAERS this could occur as a result of enhancing the gain-of-function alteration in Ca_V_3.2 channels within the cortical seizure initiation focus.

## Discussion

Heantos-4 is approved by the Vietnamese Food and Drug Administration and is used clinically to treat opiate addicts in Vietnam [4], although has not yet received formal approval for use in Western addiction treatment programs. During periods of Heantos-4 treatment, patients report not only a reduction in withdrawal symptoms and possibly craving, but also strong sedative effects. While studies are ongoing in an effort to decipher its mechanism of action with respect to drug-seeking behaviour and neurotransmitter release [5], we sought to examine its effects on burst-firing in the thalamus due to the role of this region in the control of arousal [26, 27]. We first established that Heantos inhibits Ca_V_3.1 and Ca_V_3.3 but potentiates Ca_V_3.2 channels, and subsequently found that burst-firing was differentially modulated in two distinct classes of thalamic neurons. In VB neurons that primarily express Ca_V_3.1 burst firing was inhibited by Heantos, but in TRN neurons that express a combination of Ca_V_3.2 and Ca_V_3.3 burst-firing was not affected.

### Differential modulation of T-type calcium channel isoforms

To date only a few pharmacological agents have been discovered that can selectively distinguish between the three distinct T-type calcium channel isoforms. Ca_V_3.2 channels display a degree of redox sensitivity due to the presence of extracellular cysteine residues and/or a histidine residue found only in Ca_V_3.2, but not Ca_V_3.1 or Ca_V_3.3 channels [19, 28, 29]. As a result Ca_V_3.2 channels are inhibited by oxidation and potentiated by reduction of at least one of these residues. In addition, Ca_V_3.2 displays a higher efficacy to inhibition by nickel (<100 μM) and other trace metals due to the presence of a high affinity binding and metal-catalyzed oxidation site provided by the same extracellular histidine residue [30]. Heantos-4 is a mixture of 12 organic herbs [4, 5] and the potentiation of Ca_V_3.2 could occur as a result of one or more active components. Of note, a shift in the activation curve to hyperpolarized potentials is observed in Ca_V_3.1 and Ca_V_3.2 channels, but not in Ca_V_3.3 channels. Redox modulation of Ca_V_3.2 channels induces only nominal effects on the voltage-dependence of activation [29]. However, reduction of recombinant Ca_V_3.2 channels with L-cysteine has been reported to cause an approximate 5 mV shift towards hyperpolarized potentials [19]. This is in agreement with Heantos-mediated potentiation of Ca_V_3.2 currents via reduction of redox-sensitive channel residues. However, the mechanism underlying the Heantos-mediated differential modulation of voltage-dependence of activation in Ca_V_3.1 and Ca_V_3.3 channels is unknown. It is possible that Heantos contains certain components that inhibit both Ca_V_3.1 and Ca_V_3.3, and other components that modulate the gating dynamics of Ca_V_3.1 only. Alternately, a single component of Heantos may have a differential effect on the gating of Ca_V_3.1 and Ca_V_3.3, while simultaneously inhibiting both channels. Once analysis of the active components in Heantos has been performed it will be possible to dissect out how this differential modulation occurs. Further research is required to determine the exact compound(s) that induce the potentiation of Ca_V_3.2 and also the inhibition of Ca_V_3.1 and Ca_V_3.3. To this end, we are currently conducting mass spectrographic analyses of Heantos-4 compounds that pass into the cerebrospinal fluid compartment in vivo. Preliminary analyses confirm the presence in brain CSF of > 98% of phytochemical classes identified with the components of Heantos-4 (data not shown). In the meanwhile, given that existing T-type channel pharmacological tools are limited to the redox-mediated inhibition or potentiation of Ca_V_3.2 without affecting Ca_V_3.1 or Ca_V_3.3, Heantos-4 provides a novel pharmacological tool to inhibit Ca_V_3.1 or Ca_V_3.3 and potentiate Ca_V_3.2.

### Selective inhibition of VB thalamic neurons

Agents that block all three T-type channel isoforms abolish both depolarizing burst-firing in TRN neurons and rebound burst-firing in thalamocortical neurons [18, 31–34]. Of note, to date no pharmacological agents have been identified that can selectively inhibit burst-firing in thalamocortical neurons without also inhibiting TRN neurons.

The T-type calcium currents that underlie low-threshold burst-firing in thalamic neurons have been studied extensively (for review see [8, 14]). In thalamocortical neurons the relatively fast and short burst duration supports a role of Ca_V_3.1 due to its fast activation/inactivation kinetics and hyperpolarized voltage-dependence of activation [35]. This is further supported by analysis of T-type calcium channel mRNA expression using both *in situ* hybridization [15] and quantitative PCR [17, 36]. In addition, genetic ablation of the *Cacna1g* gene that encodes Ca_V_3.1 in mice abolishes burst-firing in thalamocortical neurons [16].

In TRN neurons the burst duration activates quickly but is of longer duration than in VB neurons indicating that the combined Ca_V_3.2/ Ca_V_3.3 currents underlie the LTS since Ca_V_3.2 channels activate quickly and Ca_V_3.3 channels inactivate slowly [8, 37]. This notion is supported by mRNA analyses [15] and by redox pharmacological evidence. As discussed, Ca_V_3.2 is redox sensitive and TRN neurons display enhanced or suppressed burst-firing upon application of reducing and oxidizing agents, respectively [19]. In support, T-type currents in TRN neurons can be potentiated up to 50% by reducing agents and inhibited up to 50% by oxidizing agents. Further, in mice following genetic ablation of the *Cacna1i* gene, encoding Ca_V_3.3 the T-type current is approximately 40% of the total T-type current in TRN neurons of wild-type mice [38]. In a separate study investigating a strain of mice lacking the Ca_V_3.3 channel, T-type calcium currents were attenuated by approximately 80% in comparison to wild-type mice, although low threshold bursts could still be elicited in 75% of animals [39]. Of note, the remaining T-type calcium current is absent and burst-firing abolished in double Ca_V_3.2/Ca_V_3.3 knockout mice [39]. Taken together Ca_V_3.2 currents are predicted to contribute 20-40% of the total T-type calcium channel current in TRN neurons, with the remaining current contributed by the Ca_V_3.3 isoform. This assumption is supported by our finding that Heantos-4 has no effect on burst-firing in TRN neurons and agrees with our in vitro data showing potentiation of Ca_V_3.2 and inhibition of Ca_V_3.3 currents and predicted to result in opposing modulation that cancels out any global effect on the total T-type current.

### Heantos and T-type calcium channel blockade in addiction and neurological disorders

To date, Heantos-4 has been used clinically solely for the purpose of treating aspects of drug addiction. The paraventricular thalamic nucleus (PVN) has been implicated in drug-seeking behaviour [40] and T-type calcium channel activity has been identified in midline PVN neurons [41, 42]. While Ca_V_3.1 mRNA is expressed at higher levels in burst-firing PVN neurons than the other T-type channel isoforms, the PVN LTS is abolished by 50 μM nickel implicating Ca_V_3.2 since this concentration would not block Ca_V_3.1 currents [43]. Further, Ca_V_3.2, not Ca_V_3.1 appears to be involved in pain transduction in the PVN [44]. With further relevance to addiction, TTA-A2, a pan T-type calcium channel blocker attenuates food- versus nicotine-induced cue-potentiated reinstatement for a response previously reinforced by food administration in rats [45]. This suggests that T-type antagonists have the potential to alleviate nicotine addiction. Although limited, these studies provide support for a direct role of T-type calcium channels in the acquisition and/or maintenance of addiction.

In addition to addiction, T-type calcium channels are implicated in a number of other neurological disorders including in epileptic seizures [12–14, 46–48], anxiety [49–51], cognitive and memory impairments [52, 53], and in the transmission of pain [54–57]. Pan T-type blockers that block burst-firing in both VB [18] and TRN [31] neurons have been shown to suppress seizures in animal models of absence epilepsy [31, 58], prevent seizure kindling in a model of complex-partial seizures [59], and suppress tonic-clonic seizures [60]. In terms of pain signalling, pan T-type antagonists have shown efficacy in reducing pain sensation in both animal models [54, 61–63] and humans [64, 65]. T-type calcium currents underlying burst-firing and slow oscillations in the thalamocortical system have been extensively linked to non-REM sleep [6]. Somewhat counterintuitively, pan-T-type calcium channel antagonists have been shown to induce sedation [66, 67] rather than the awake state, perhaps indicating that the T-type calcium channel subtypes individually mediate distinct roles within the thalamocortical system and that block of all three subtypes shifts equilibrium to the sleep state. Taken together, Heantos-4 may have potential as a subtype-specific T-type calcium channel antagonist for use as both a pharmacological tool and potential therapeutic agent.

In summary, Heantos-4 is a clinically utilized treatment for drug addiction and is currently the subject of studies aimed at determining its mechanism(s) of action [5]. Here we show that Heantos-4 selectively inhibits Ca_V_3.1 and Ca_V_3.3 but potentiates Ca_V_3.2 T-type calcium currents. In addition, Heantos-4 selectively inhibits burst-firing in VB thalamic neurons while having no significant effect on TRN neurons. This supports the data from exogenously expressed T-type calcium channel isoforms and their corresponding differential expression in distinct VB and TRN thalamic nuclei. That Heantos-4 appears to exacerbate absence seizure activity in GAERS suggest that caution may be warranted in the clinical usage of Heantos-4 in seizure-prone individuals. Determining whether the effect of Heantos-4 on T-type calcium channels is related to its role in the treatment of drug-craving and withdrawal will require additional studies. Regardless, our findings that Heantos-4 differentially modulates distinct neuronal populations expressing T-type calcium channel isoforms may be relevant with respect to a number of neurological disorders in which T-type calcium channels are implicated [13, 68].

## Methods

### Electrophysiology on exogenously expressed T-type calcium channel isoforms

Flp-In 293 cells (Invitrogen), stably expressing pcDNA5/FRT plasmids containing hCa_V_3.1, hCa_V_3.2 or hCa_V_3.3 were grown at 37 °C in DMEM supplemented with 10% heat inactivated fetal bovine serum. Cells expressing T-type calcium channel isoforms were selected by incubation with hygromycin. Cells were seeded on poly-D-Lysine (0.1 mg/ml) coated glass coverslips and hygromycin removed from media 48 h before voltage clamp recordings.

Calcium currents were recorded at 22–24 °C using whole-cell voltage clamp with the following solutions containing in mM: Internal: 120 Cs-Methanesulphonate, 11 EGTA, 10 HEPES, 2 MgCl_2_, 5 MgATP and 0.3 NaGTP (pH 7.2) External: 2 CaCl_2_, 1 MgCl_2_, 10 HEPES, 40 TEACl, 92 CsCl and 10 Glucose (pH 7.4). Fire polished patch pipettes (borosilicate glass) had typical resistances of 3 to 5 MΩ when containing internal solution. The recording chamber was grounded with a Ag/AgCl pellet. Whole-cell currents were recorded at room temperature using an Axopatch 200B amplifier (Axon instruments Inc., Union City, CA). Data was acquired with pClamp software package version 9 (Axon Instruments Inc.). Series resistance (Rs) was compensated by 65–75% and seals with Rs values higher than 20MΩ or cells with peak current lower than 100pA were discarded. Currents sampled at 10 kHz and filtered at 2 kHz. Data analysis was carried out using Clampfit 9 (Axon Instruments Inc.) and software Origin version 7.5 (OriginLab Corp., Northampton, MA).

Timecourse of drug action was obtained by depolarizing the membrane with a 200 msec pulse to −30 mV from a holding potential of −100 mV every 5 s. The peak calcium current was taken from each trace for analysis. A two minute stable baseline was acquired before initiating a five minute Heantos application via the perfusate.

The current-voltage (I-V) relationship was obtained before and after the timecourse protocol by depolarizing the membrane with pulses (Ca_V_3.1 and Ca_V_3.2 = 150 msec, Ca_V_3.3 = 450 msec) from a holding potential of −110 mV. Test pulses from −90 to +10 mV were applied at 5 mV steps. Peak amplitude of calcium currents was plotted against test pulse potential and I-V curves were fitted using a modified Boltzmann equation: I = (Gmax*(Vm-Er))/(1 + exp((Vm-V50)/k)), where Gmax is the maximum value of membrane conductance, Vm is the test potential, Er is the extrapolated reversal potential, V50 is the half-activation potential, and k (Slope constant: k = RT/zδF; where R = gas constant, T = absolute temperature, z = valence of conducting ion, δ = electrical distance across the membrane, F = Faraday’s constant) reflects the voltage sensitivity. Activation curves were obtained by calculating conductance from the I-V curves and plotting the normalized conductance as a function of the membrane potential. The data was fitted with the Boltzmann equation: G/Gmax = A2 + (A1-A2)/(1 + exp((Vm-V50)/k)), where A1 is minimum normalized conductance, A2 is maximum normalized conductance, Vm is the test potential, V_50_ is the half-activation potential, and k value the slope of the activation curve (Slope constant).

### Acute brain slice electrophysiology

Male and female Wistar rats (P15–P20) were used in acute brain slice experiments in accordance with Canadian Council for Animal Care guidelines.

Animals were anesthetized using isoflurane (5% in O_2_), sacrificed by decapitation, the brains rapidly removed and transferred to ice cold sucrose-aCSF containing in mM: 214 sucrose, 26 NaHCO_3_, 1.25 NaH_2_PO_4_, 11 glucose, 2.5 KCl , 0.5 CaCl_2_, 6 MgCl_2_, bubbled with 95% O_2_:5% CO_2_. Brain tissue was glued to a cutting chamber in a vibrating microtome (VT 1200, Leica, USA), which was then filled with ice cold sucrose-aCSF. Horizontal brain slices containing the whole thalamus (350 μm thick) were cut from the level of the ventral TRN/VB and incubated for a minimum of 30 min at 34 °C in a current clamp recording solution containing in mM: 126 NaCl, 2.5 KCl, 26 NaHCO_3_, 1.5 NaH_2_PO4, 2 CaCl_2_, 2 MgCl_2_, 10 glucose; bubbled with 95% O_2_:5% CO_2_. Slices were transferred to a recording chamber superfused with current clamp recording solution and maintained at 33–35 °C. VB and TRN neurons were visualized using a DIC microscope (Axioskop 2-FS Plus, Carl Zeiss) and infrared camera (IR-1000, DAGE MTI) and visually identified by their location, morphology and orientation. All recordings were undertaken using a Multiclamp 700B amplifier and pClamp software version 9 (Molecular devices). The recording chamber was grounded with a Ag/AgCl pellet.

Whole cell current clamp recordings were undertaken using fire polished borosilicate glass pipettes (4–6 MΩ) filled with the following solution containing in mM: 120 K-gluconate, 10 HEPES, 1 MgCl_2_, 1 CaCl_2_, 11 KCl, 11 EGTA, 4 MgATP, 0.5 NaGTP , pH adjusted to 7.2 using KOH, osmolarity adjusted to 290 mOsm/kg using D-mannitol. The liquid junction potential for current clamp solutions was calculated as +13.3 mV and corrected off-line. To evaluate input–output neuronal responses to hyperpolarization and depolarization, DC current was injected from −100 pA to +200 pA in 10 pA increments for a duration of 1200 ms at the neuron’s intrinsic resting membrane potential. VB neurons that did not rebound burst fire with ≤ −100 pA current injection were then evaluated with a current injection protocol where DC current was injected from −100 pA to −500 pA in 50 pA increments. Membrane potential responses under current clamp conditions were sampled at 50 kHz and filtered at 10 kHz. Bridge balance was monitored during recordings and any neurons displaying bridge balance values greater than 25 MΩ were discarded. Capacitance neutralization was performed between 3.8 and 4.2 pF.

### EEG recording

Adult GAERS (4–6 month old) were anesthetized with isoflurane and implanted with skull screw electrodes on the somatosensory cortex surface (bregma = +1.2 mm, lateral = ± 5.0 mm, depth below skull = 0.1 mm) and a reference electrode in the cerebellum (lambda = -0.5 mm, lateral = -0.5 mm, depth below skull = 1 mm). Electrodes were connected to a custom EEG interface implanted on the skull. Following a 2 week recovery period the interface was connected to a wireless headstage (W2100, Multichannel Systems, Germany) and EEG signals acquired in the animals’ home cage for 1 h.

### Drugs

Heantos-4, a brown, tea-like powder was provided by Dr. Sung at the Institute of Chemistry, Vietnam Academy of Science and Technology (Hanoi, Vietnam). For in vitro electrophysiology experiments Heantos-4 was prepared at 1 mg/ml by immersing in aCSF, vortexing for several seconds and incubating at 50 °C for 10 min followed by sonication at 22–24 °C for 5 min. Insoluble components of Heantos were then filtered and the flow-through used in all experiments at this concentration (1 mg/ml) or as a serial dilution.

For in vivo experiments Heantos-4 was mixed into 0.5% carboxymethylcellulose (CMC) in 0.9% saline and administered via oral gavage 30 min prior to EEG recording.

### Statistics

Data followed a normal distribution and statistical significance was calculated using Student’s T-test (paired where appropriate) or ANOVA with Tukey's Post-hoc test taking *P* value < 0.05 as significant. Data was plotted as mean values ± standard error.

## Abbreviations

Ca_V_: Voltage-gated calcium channel
GAERS: Genetic Absence Epilepsy Rats from Strasbourg
HVA: High Voltage-Activated
LTS: Low Threshold calcium Spike
SWDs: Spike-Wave Discharges
TRN: Reticular thalamic nucleus
VB: Ventrobasal thalamic nucleus
